# Independent evaluation of machine learning and deep learning models for breast cancer detection

**DOI:** 10.1371/journal.pdig.0001747

**Published:** 2026-09-28

**Authors:** Deepthi, Ashalatha Nayak, Rani Oomman Panicker

**Affiliations:** Manipal Institute of Technology, Manipal Academy of Higher Education, Manipal, India; The Ohio State University, CHINA

## Abstract

Breast cancer is one of the leading causes of cancer-related mortality worldwide, and despite advances in clinical diagnosis, challenges such as inconclusive imaging results and inter-observer variability highlight the need for complementary computational approaches to support early and accurate detection. In this study, we provide an overview of several machine learning (ML) and deep learning (DL) algorithms used for breast cancer detection with structured clinical data and imaging data. We applied ML models to structured clinical data which included Wisconsin Breast Cancer Dataset, CSAW-CC dataset and SEER database using Random Forest, Decision Tree, Logistic Regression, Gradient Boosting, and Bayesian classifier models. DL models, specifically Convolutional Neural Networks (CNN), ResNet50, VGG16, and DenseNet, were utilized in mammography, histopathology, and ultrasound datasets. The experimental results show that the DL models outperformed ML models in image classification tasks, obtaining accuracy rates of 97.88% for histopathological images and 92.00% for ultrasound data. However, ensemble-based and probabilistic ML models achieved strong performance on structured clinical data, with predictive accuracy of up to 98%. All performance metrics are reported with 95% confidence intervals.Model calibration was assessed using the Expected Calibration Error (ECE) and reliability diagrams. Logistic Regression, Gradient Boosting, and Random Forest produced the best-calibrated probability estimates among the structured-data classifiers, while the transfer-learning-based deep convolutional architectures were well calibrated on the histopathology imaging datasets. Calibration was comparatively weaker for Decision Tree and Naive Bayes among the structured-data classifiers, as well as for the from-scratch CNN baseline and some deep learning models evaluated on the smaller BUSI dataset. The results indicate that model performance is significantly influenced by data modality and that artificial intelligence may serve as a valuable computational tool to support clinical breast cancer diagnosis workflow. Further prospective validation using clinical data across diverse patient populations will be essential to confirm the robustness, interpretability, and generalizability of these findings.

## Author summary

Breast cancer is one of the major contributors to cancer deaths in the world, and early diagnosis has a critical role in improving the survival rate of the patients. This study evaluates the effectiveness of artificial intelligence method across different types of medical data used in breast cancer diagnosis. We used standard machine learning models in structured clinical data and deep learning models in medical images such as histopathology slides, ultrasound scans and mammograms from publicly-available repositories. All datasets underwent standardized preprocessing, model training and evaluation using standard performance measures. We find that no particular approach outperforms others when working with all types of data: conventional machine learning was more successful in the classification of tabular clinical data, but deep learning models were more accurate in the classification of imaging data. These results show that the best choice of models is determined by the characteristics of the data and not only by the complexity of the models. This study supports the potential of artificial intelligence as an assistive tool for breast cancer diagnosis but emphasizes the need for further validation in clinical settings.

## Introduction

Cancer is a class of diseases characterized by unchecked cell proliferation that in turn causes nearby tissue invasion as well as spread to distant organs by way of the blood or lymphatic system. Among all cancer types, breast cancer remains the most frequently diagnosed worldwide and continues to represent a major global public health issue [1]. The disease originates from atypical growth within breat tissue; although it predominantly affects women, it can also occur in men, who possess breast tissue as well. Tumor growth is a result of breakdown of regulatory processes which control cell division and the degree of disease advancement which in turn plays a role in what treatment is used and how the disease will progress in the affected person.

Clinically breast cancer is characterized by the anatomic site of origin and the pathologic features of the malignant cells. Common subtypes include lobular carcinoma, ductal carcinoma in situ (DCIS) and invasive ductal carcinoma [2]. In many cases malignancy originates in the milk ducts and may either remain localized or invade surrounding tissue. Each subtype differs considerably in biological behavior, aggressiveness and clinical outcome. Rarer but highly aggressive forms include triple negative breast cancer, inflammatory breast cancer, and Paget’s disease of the breast. These present additional diagnostic and therapeutic challenges due to their rapid progression and limited response to standard treatment [2]. For these reasons, proper diagnosis, prognosis evaluation, and treatment planning depend on appropriate classification.

Early detection of breast cancer plays a critical role in improving survival outcomes and reducing mortality. Methods for diagnosis and screening include the use of mammograms, tissue biopsies, MRI, ultrasound, and immunohistochemical reviews [3].These tools have contributed to lowering the death rate from breast cancer. Current diagnostic methods have certain limitations, as mammography can produce non-diagnostic or false-positive results that may lead to unnecessary anxiety, additional diagnostic procedures, or delays in treatment [4]. Biopsy is the gold standard for diagnosis, but its invasive nature makes it impractical for use at a population-screening level, which motivates investigation of computational tools that can complement, but not replace, established clinical diagnostic pathways.

Various machine learning strategies have been utilized for prediction and classification of diseases within the domain of structured medical data. Random Forest, Decision Tree, Logistic Regression, Gradient Boosting, and Naive Bayes have all shown positive results when used for clinical data, lab data and diagnosis data [5–7]. Due to the nature of the data being analyzed, these strategies are very effective at providing accurate and dependable results. In clinical environments, the consistent application of these strategies has enhanced the level of decision making and diagnostic accuracy.

Deep learning is a powerful way to examine unstructured data, especially medical data. With deep neural networks, complex patterns can be learned from unstructured imaging data and be associated with particular diseases [8,9]. In breast cancer research, deep learning models such as Convolutional Neural Networks (CNNs), VGG16, ResNet50, and DenseNet have shown impressive results in the analysis of the various modalities including, mammography, histopathology, and ultrasound data [10,11]. These models are especially efficient at identifying minute details in imaging data that would be challenging to find with a manual analysis.

We employed a variety of publicly available datasets in various clinical settings to improve the study’s strength and external validity. The imaging datasets that are often used to diagnosis breast cancer include the Histopathological dataset, the BreakHis dataset, and the BUSI ultrasound pictures [12,13]. Additionally, the Wisconsin Breast Cancer dataset, the CSAW-CC dataset, and the SEER dataset were rich in clinical and epidemiological data that could be utilized to predict the data through individual modeling of the data sets provided by the study’s database [14]. This approach enabled a more detailed comparison across diverse patient and data groupings.

Despite using standard machine learning and deep learning methods, the study adopts a distinctive analytical approach across a broad array of breast cancer datasets, spanning structured patient records and various medical imaging formats. This approach consistently evaluates the relationship between dataset attributes and outcome measures. The results are based on empirical experiments rather than conjecture about increased model complexity leading to improved results.

Increased complexity does not always translate to better results. Instead, results depend on the modality of the dataset. For structured tabular datasets such as the Wisconsin Breast Cancer data set, simple machine learning models tend to achieve high accuracy results due to the simple numerical attributes. However, for image-based datasets such as histopathological and mammography images, complex deep learning models are required for identifying complex features in the data. This indicates that the results are more dependent on the dataset rather than the complexity of the model. A comparative analysis will be conducted in this study to identify the most efficient models. This study follows a retrospective computational design in which all datasets contain pathology-confirmed labels, and models classify known diagnosis rather than making new clinical predictions. The clinical implications of these results should therefore be interpreted with caution, as prospective and multi-center validation will be essential before any findings can be translated into clinical practice.

The validation and training of models on a variety of datasets exhibit greater generalization and lower bias. The research addresses the main problems of processing medical data such as the uniformity of classes, noise in images, and variable quality of data used in the study [15]. The present study intends to provide some insight into the possible strengths and weaknesses of the machine and deep learning methods in breast cancer detection through a comprehensive study of different algorithms and data sets to assist in choosing appropriate methods of computation.

The aforementioned studies form the basis for this study. However, most of the existing studies use a single dataset or type of data, providing a narrow scope on the performance of algorithms in various clinical settings. This study uses a single comparison framework to examine breast cancer detection across several structured and imaging datasets.This research’s primary contributions can be summarized as follows:

1) A standardized evaluation framework is proposed for six publicly available breast cancer data sets, three for structured data and three for image data, to facilitate uniform preprocessing and performance evaluation.
2) A cross-modality evaluation between classical machine learning and deep learning models is carried out to assess the effectiveness of these models on tabular and image data.
3) An empirical analysis investigates the link between model complexity and predictive performance, showing that accuracy improvements depend on dataset characteristics rather than model sophistication alone.
4) Model robustness and generalization are also assessed across various clinical and image-based environments.
5) The research has provided significant insight in selecting the best computational models for breast cancer diagnosis.
6) A dedicated analysis of ensemble-based and probabilistic machine learning models on structured clinical datasets is presented, explaining their theoretical foundations and relative advantages.
7) A calibration analysis using the Expected Calibration Error (ECE) and reliability diagrams is presented for all classical machine learning and deep learning models, providing insight into the reliability of predicted probabilities in addition to discrimination performance.

The rest of the paper is structured as follows. An overview of relevant research on deep learning and machine learning systems for breast cancer detection is provided in the Literature Review section. The Methodology part covers the data set, preprocessing, construction, and evaluation measurement. The Results section contains the findings from the experiment and comparative analysis. The results are discussed in relation to clinical and computational factors in the Discussion section. An overview of the findings and potential future research is given in the Conclusion section.

## Related work

Many studies examined the computational techniques for identifying breast cancer utilizing both medical imaging and structured clinical data. The majority of initial studies employed datasets such as the Wisconsin Breast Cancer collection and relied on traditional machine learning algorithms. Islam et al. [10] tested various classifiers and noted that the best results were obtained with the Random Forests and Support Vector Machines. Other researchers like Ak et al. [16] and Chaurasia and Pal [17] have proved that kernel based and ensemble approaches generally outperform simple approaches such as K-Nearest Neighbour simply by choosing the right features. Preprocessing, parameter adjustment, and validation are crucial for producing accurate predictions, according to other studies by Sakib et al. [9], Ara et al. [18], and Khan et al. [19].

Subsequently, ensemble approaches were used to increase stability. When compared to individual classifiers, ensemble classifiers can produce more reliable diagnostic and prognostic results, as demonstrated by Naseem et al. [20]. Alhasani et al. [11] and Al-Imran et al. [14] both stress that there isn’t a single algorithm that consistently performs well for every metric when taking the larger picture into account. According to Sweetlin and Saudia [21], the selection of the dataset and the feature engineering process have a big impact on the results. The majority of machine learning research only test on one structured dataset, therefore the outcomes may not necessarily hold up for a different collection of data, even when certain aspects of studies indicate great accuracy.

Deep learning techniques, particularly convolutional neural networks, have become increasingly popular as digital medical imaging has grown. A baseline for image-based categorization is provided by the BreakHis histopathology dataset, which was first presented by Spanhol et al. [7]. While Mohanakurup et al. [13] suggested a composite dilated backbone network to capture spatial characteristics, Das et al. [12] used this dataset to create an ensemble deep learning approach. Singh and Kumar [22] adopted a contrary approach. They used inception and residual modules to enhance their performance on histopathological images. Similar approaches have been reported by Boumaraf et al. [15] Reshma et al. [23] and Alhsnony and Sellami [24]. Their findings showed that the deep learning models generally outperform the traditional machine learning methods when dealing with image data due to its ability to learn complex visual patterns

Researchers have also applied computational techniques across several imaging modalities. For instance, Yadav and Jadhav [25] used machine learning and thermal infrared pictures. According to their findings, this combination may be useful for non-invasive diagnostics. While Dar et al. [26] addressed issues such dataset limits, overfitting, and inconsistent validation, Hamed et al. [27] examined deep learning architectures for breast cancer diagnosis. The key trends, such as convolutional architectures, transfer learning, and ensemble techniques, were compiled in surveys by Chugh et al. [28] and Nemade et al. [29]. A comparative study of machine learning and deep learning techniques was carried out by Fatima et al. [30], although their study was restricted to a subset of datasets without a unified evaluation framework.

The majority of current research is focused on either clinical data or a particular type of image, despite repeated findings of high prediction accuracy. This limits cross-study comparison of results due to variations in data pre-processing, performance measurements, and testing procedures.Furthermore, few studies compare the performance of models on multiple datasets using metrics like AUC-ROC, accuracy, precision, and recall. This has therefore hindered the evaluation of various data types, and there is a strong need to investigate the potential of the computational approaches systematically in order to realize their full capabilities for breast cancer diagnosis.

## Materials and methods

### Study design

This study used a retrospective computational benchmarking approach to assess the capability of machine learning and deep learning algorithms in the detection of breast cancer across both structured and imaging data modalities.All datasets were obtained from publicly available repositories and have retrospective, pathology validated labels, thus models predict known outcomes rather than prospective or clinically validated predictions. The study process consists of data collection, data pre-processing, model implementation, training, and evaluation. Multiple models were used on structured data and image data based on their data structures. This study also highlights the comparison of different algorithms on different datasets to find the most reliable and accurate methods.

### Datasets

All datasets included in this study were obtained from publicly accessible sources, and therefore, ethical approval and patient consent were not required.These datasets are retrospectively collected and contain pathology-confirmed labels; the models therefore classify known diagnoses rather than making prospective clinical predictions. A summary of each dataset is provided in Table 1. It should be noted that information on breast density was not available in any of the datasets used in this study; this is acknowledged as a limitation in the Limitations section.

**Table 1:** Summary of the datasets used in this study.

Dataset	Modality	Key Characteristics	Role in the Proposed Research
SEER Breast Cancer Dataset	Clinical registry	Population-based dataset including demographic, pathological, and survival variables	Survival modeling and diagnostic prediction using machine learning algorithms
CSAW-CC Mammography Dataset	Mammography imaging	Screening mammograms requiring preprocessing and lesion feature extraction	Binary tumor classification using feature-engineered ML models
Wisconsin Breast Cancer Dataset (WBCD)	Cytological tabular data	569 instances described by 30 numerical tumor attributes	Benchmark evaluation of classical classifiers for benign–malignant prediction
Breast Histopathology Images Dataset	Microscopic RGB images	High-resolution tissue patches capturing cellular morphology and tumor patterns	Deep learning-based cancerous versus non-cancerous tissue classification
BreakHis Dataset	Multi-magnification histopathology images	7,909 images covering benign and malignant tumor subtypes	Binary and multi-class subtype classification using convolutional neural networks
BUSI Dataset	Ultrasound imaging	780 labeled images (normal, benign, malignant) with speckle noise characteristics	Multi-class tumor classification to evaluate convolutional neural network robustness

#### Wisconsin breast cancer diagnostic (WBCD) dataset.

The WBCD dataset [21] contains 569 instances described by 30 numeric features computed from digitized fine-needle aspirate (FNA) images of breast masses, labeled as benign (357 instances, 62.7%) or malignant (212 instances, 37.3%). No missing values were present in this dataset. It serves as a benchmark dataset for evaluating classical classifiers on small, focused structured data. Demographic information such as patient age and ethnicity is not included in this dataset. The dataset is retrospectively collected from clinical records.

#### SEER breast cancer dataset.

Population-level clinical and survival data were obtained from the Surveillance, Epidemiology, and End Results (SEER) Program [19], comprising 4,024 patient records with demographic, pathological, and treatment variables. The target variable was survival status (alive vs. deceased). The class distribution was approximately 84.8% alive and 15.2% deceased, which is representative of the class imbalance in survival datasets. The dataset includes demographic variables such as age at diagnosis, race, and marital status. However, the dataset has been retrospectively collected and reflects historical patterns of clinical practice.

#### CSAW-CC mammography dataset.

The CSAW-CC dataset [17,23] includes mammogram images from the screening program at Karolinska University Hospital. In this work, handcrafted radiomics and texture features were extracted from the mammography images and used as structured input for classical ML classifiers, allowing for a fair comparison with other tabular datasets. After the pre-processing stage, the dataset contained 1,944 instances with binary labels (malignant or not). Detailed information on patient demographics is not publicly available for this dataset. The mammographic images contain cases with subtle and overlapping features and different levels of lesion complexity. The data set was obtained retrospectively from a clinical screening program.

#### Breast histopathology images dataset.

The dataset consists of 277,524 50×50 pixel patches extracted from whole-slide images of invasive ductal carcinoma (IDC) specimens. Each patch is classified as IDC-positive (malignant) or IDC-negative (non-cancerous). Due to computational constraints, we used a stratified subset of 10,000 images for model training and evaluation, maintaining the original class ratio (approximately 59.5% negative and 40.5% positive). The patches show different degrees of lesion complexity, such as overlapping cellular structures and different staining intensities. Demographic information on the patients is not available in this dataset. All images were collected retrospectively from pathology archives.

#### BreakHis dataset.

The BreakHis dataset [7,17,23] contains 7,909 microscopic histopathological images from 82 patients at four magnification levels (40×, 100×, 200×, 400×). Images are labeled as benign (2,480 images, 31.4%) or malignant (5,429 images, 68.6%), reflecting a notable class imbalance. The subtypes of benign cancers include: adenosis (A), fibroadenoma (F), phyllodes tumor (PT), and tubular adenoma (TA). The malignant subtypes of histopathological cancers in the BreakHis data set include: ductal carcinoma (DC), lobular carcinoma (LC), mucinous carcinoma (MC) and papillary carcinoma (PC). The complexity of the individual lesion classifications from the BreakHis dataset are enhanced due to the diversity of classifications as well as the significant differences in the appearance of each tumor classification based on varying levels of magnification. In addition, details about patient demographics (over the 82-patient reflected in the dataset) are not included in the publicly available data and that only image-level identifying information is available, as all images will be classified based on retrospective retrieval from pathology records regardless of whether or not their clinical diagnoses is proven to meet the requirements of a correct histopathological diagnosis. All images were included in this study regardless of magnification level. In addition, the patient identifier number was used to split patient data between the training and testing data subsets to ensure that data were not used in the training dataset portion when conducted the train/test split, in order to minimize potential for dataset leakage between training and testing [17,23].

#### Breast ultrasound images (BUSI) dataset.

BUSI dataset [27,30] consists of 780 ultrasound images acquired from 600 female patients with ages ranging between 25–75 years in 2018. The images have been classified into either normal (133 images), benign (437 images), or malignant (210 images). In the binary classification task, normal and benign images were categorized together as one category making 570 non-malignant images and 210 malignant images. This data has varying complexity levels where the lesions include irregular margins, posterior acoustic shadowing, and speckle noise. This data set is retrospective in nature since it has been acquired from clinical records.Ultrasound imaging is often utilized as a non-invasive screening tool for detecting breast cancer in young women with dense breast tissue [25,28].

### Data preprocessing

Depending on the kind of feature, statistical imputation approaches such as mean, median, or mode imputation were used to fill in the missing values in the tabular datasets. To guarantee that the range of all features is uniform, feature scaling or normalizing was also applied. This is particularly important for feature-range-sensitive models, such as logistic regression. Label encoding or one-hot encoding were used to handle categorical variables. Stratified sampling was used to separate the datasets into training and testing sets in order to eliminate bias [20,29]. For all structured datasets, an 80:20 train-test stratified split was applied, with 20% of the training portion further held out as a validation set. For image-based datasets, a 70:15:15 (train-validation-test) stratified split was used. To ensure reproducibility, a fixed random seed of 42 was used across all experiments.

The preprocessing of image-based datasets included resizing each image to 224 × 224 pixels, the standard input resolution for VGG16, ResNet50, and DenseNet architectures. Pixel values were normalized to the range [0, 1] by dividing by 255. Data augmentation techniques including random rotation (up to ±15°), horizontal and vertical flipping, scaling (zoom range 0.1), and brightness adjustment (±10%) were applied exclusively within the training set to avoid data leakage into the validation or test sets. No augmentation was performed on validation or test data. These augmentation techniques help build more robust models with improved generalization. Subsequently, the datasets were divided into training, validation and test datasets to allow objective evaluation of the models [22].

### Model implementation

The classical machine learning algorithms that have been implemented for tabular data are Logistic Regression, Decision Trees, Random Forests, Naive Bayes Classifiers, and Gradient Boosting [18,24]. These algorithms function well with structured data and have been demonstrated to be successful in breast cancer prediction in previous works.

Deep learning models were used for image classification tasks. The baseline models were Convolutional Neural Networks (CNN), and pre-trained models such as VGG16, ResNet50, DenseNet121, and DenseNet201 were used for transfer learning. The pre-trained models were refined on the breast cancer datasets to improve feature extraction and training time for subtle features in histopathological and ultrasound images [23–25].

Deep learning models were implemented using Python 3.10, TensorFlow 2.12, and Keras 2.12 running on a system with an NVIDIA RTX 3050 GPU (8 GB VRAM), 16 GB RAM, and CUDA 11.8.

### Ensemble-based and probabilistic machine learning models

Among the classical machine learning approaches evaluated in this study, ensemble-based and probabilistic models stand out for their consistently strong performance on structured clinical datasets, and are discussed in detail in this section. This section outlines the theoretical basis and practical advantages of these model families in the context of breast cancer classification.

#### Ensemble-based models.

Ensemble methods improve classification performance by aggregating predictions from multiple base learners, thereby reducing the variance and bias that typically affect individual models. Two ensemble strategies were employed in this study: bagging, represented by Random Forest, and boosting, represented by Gradient Boosting.

Random Forest builds a collection of decision trees, each trained on a bootstrap sample drawn from the training data, with a randomly selected subset of features considered at each split. For classification tasks, predictions from individual trees are combined using majority voting. When randomness is deliberately introduced during tree construction, the correlation between individual trees is reduced, which lowers the overall prediction variance without significantly raising bias [20]. As the number of trees in the forest increases, the generalization error converges to a stable value determined by the strength of the individual trees and the correlation among them. Due to this characteristic, Random Forest is especially well suited for large, structured clinical datasets with complex interactions among therapeutic, pathological, and demographic variables, such as the SEER dataset.

Gradient Boosting takes a different approach by constructing trees sequentially. Each successive tree is trained to solve the residual errors of the current ensemble, thereby iteratively reducing the overall prediction bias. This sequential error correction makes Gradient Boosting particularly effective when the underlying classification boundary is complex and non-linear, as typically is the case in clinical data with mixed variable types. Both Random Forest and Gradient Boosting make relatively few distributional assumptions about the input data, which contributes to their robustness across diverse structured datasets.

#### Probabilistic models.

The Naive Bayes classifier is based on the Bayes theorem and calculates the posterior probability of each class given the observed feature values. Given the class label, the features are assumed to be conditionally independent. This allows the joint likelihood to be expressed as the product of the likelihoods of each individual feature. However, this independence assumption is rarely true in practice, the Naive Bayes classifier has been shown to be competitive in a wide range of real-world classification tasks, especially when the training data are scarce or the feature correlations are moderate [29].

In this investigation, Naive Bayes exhibited the best level of accuracy (96.00%) compared to other ML algorithms when applied to the Wisconsin Breast Cancer Diagnosis Data Set, whose small data size (569 observations) and medium correlations between variables match the algorithm’s assumptions. In contrast, with datasets that have greater feature space and high variable correlation, like the CSAW-CC mammography features, the independence assumption becomes a limiting factor and ensemble methods tend to outperform Naive Bayes.

#### Practical considerations.

From a clinical decision support perspective, the choice between ensemble and probabilistic models depends on both the dataset characteristics and the deployment context. Ensemble methods are generally preferred for large, well-structured clinical registries where complex variable interactions are expected, as demonstrated by their strong performance on the SEER dataset in this study. While the probabilistic models exhibit computational efficiency and well-calibrated probabilities, which may be advantageous in resource-constrained or real-time screening settings. Compared with deep neural networks, both model families offer greater transparency in their decision processes, which is an important consideration for clinical adoption and regulatory compliance.

### Training strategy and optimization

The models were optimized using adaptive learning strategies, namely the Adam optimizer with mini-batch gradient descent. The initial learning rate was set to 1e-3 for the warm-up phase and reduced to 1e-5 during fine-tuning. Batch size was set to 32 for all deep learning experiments. Training was performed for a maximum of 100 epochs, with early stopping applied based on validation loss with a patience of 10 epochs. Furthermore, to avoid overfitting and enhance generalization, L2 weight regularization (weight decay = 1e-4), dropout (rate = 0.5 on fully connected layers), and early stopping were applied [20,27]. For classical ML models, hyperparameters were selected via 5-fold cross-validation grid search on the training set, using the following grids: for Random Forest, $n_{estimators}\in \{100,200,500\}$ and max_depth∈{None,10,20}; for Gradient Boosting, $n_{estimators}\in \{100,200\}$ and learning_rate∈{0.05,0.1}; and for Logistic Regression, C∈{0.01,0.1,1,10}. All reported test-set metrics were computed on the held-out test set only, after hyperparameter selection was complete.

The performance of the models has been evaluated by employing the typical classification metrics such as the area under the receiver operating characteristic curve (AUC), accuracy, precision, recall, and F1-score. To evaluate the classification result, a confusion matrix was used, and cross-validation was used to improve the reliability of the assessment [22,24,29].

To quantify uncertainty, 95% confidence intervals (CIs) were calculated for both accuracy and AUC-ROC. The corresponding confidence intervals are reported in Tables 3, 5, 7, 10, 12, and 14, which accompany the performance results for each dataset. For datasets with large test sets (SEER, n = 805; CSAW-CC, n = 389; Breast Histopathology, n = 1,500; and BreakHis, n = 1,187), the confidence intervals were estimated using the normal approximation method, as defined in Equation (1). Equation 1:


$$\begin{array}{c} CI=\hat{p}\pm 1.96\times \sqrt{\frac{\hat{p}(1-\hat{p})}{n}} \end{array}$$ (1)

where $\hat{p}$ is the observed accuracy or AUC-ROC and *n* is the test set size. For datasets with small test sets (WBCD, n = 114; BUSI, n = 117), the Wilson Score interval was used instead, as the normal approximation can produce upper bounds exceeding 100% when sample sizes are small and metric values are high, making it statistically inappropriate. Statistical comparisons between the best-performing models per dataset were performed using McNemar’s test for paired classifiers, with a significance threshold of p < 0.05.

### Calibration analysis: Expected calibration error and reliability diagrams

Discrimination metrics such as accuracy and AUC-ROC describe how well a model separates classes, but they do not indicate whether the predicted probabilities themselves are trustworthy. This distinction is particularly relevant in a clinical decision-support context, where a predicted probability of malignancy is only useful if it approximates the true likelihood of malignancy. To assess this, we additionally evaluated the calibration of every classical machine learning and deep learning model using the Expected Calibration Error (ECE) and reliability diagrams [31,32].

For a given classifier, the predicted probabilities for the positive (malignant) class on the held-out test set were partitioned into *M* = 10 equal-width bins spanning the interval [0,1]. Within each bin $B_{m}$, the mean predicted confidence $conf(B_{m})$ and the observed empirical accuracy $acc(B_{m})$ (the fraction of samples in that bin whose true label was the positive, malignant, class) were computed. The ECE is then the sample-weighted average absolute difference between confidence and accuracy across all bins, as given in Equation 2:


$$\begin{array}{c} ECE=\sum_{m=1}^{M}\frac{\left|B_{m}\right|}{n}\;|acc(B_{m})-conf(B_{m})| \end{array}$$ (2)

where *n* is the total number of test samples and $\left|B_{m}\right|$ is the number of samples falling into bin *m*. An ECE of 0 indicates perfect calibration, whereas larger values indicate greater divergence between predicted confidence and observed outcomes. For each model, a reliability diagram was generated, plotting observed accuracy against mean predicted confidence for each bin, alongside the line of perfect calibration (*y* = *x*). The distribution of test samples across the confidence bins was examined for every model to assess the reliability of the calibration estimates. Bins containing only a small number of samples were identified because their observed accuracies may be less reliable. To keep the manuscript concise, the corresponding histograms are not shown alongside the reliability diagrams in Figs 1–Fig 6. Instead, their impact on the interpretation of the calibration results is discussed qualitatively where sample size is an important consideration, such as for the BUSI dataset in the Results and Discussion section.

**Fig 1:**
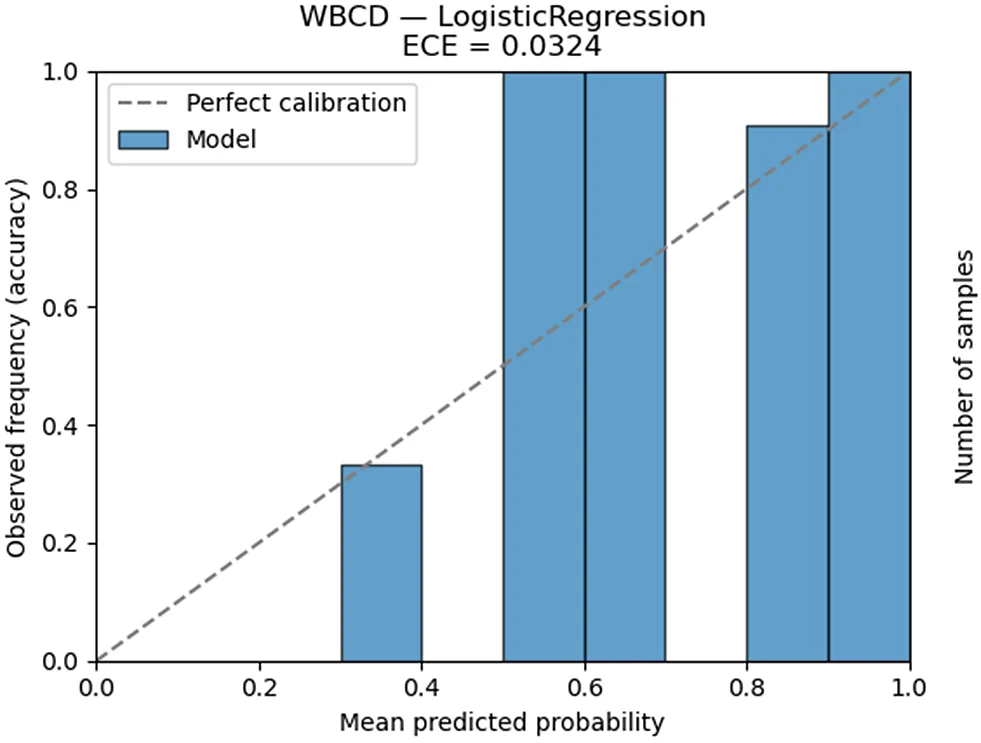
Reliability diagram for the best-calibrated classifier on the WBCD dataset (Logistic Regression).

**Fig 2:**
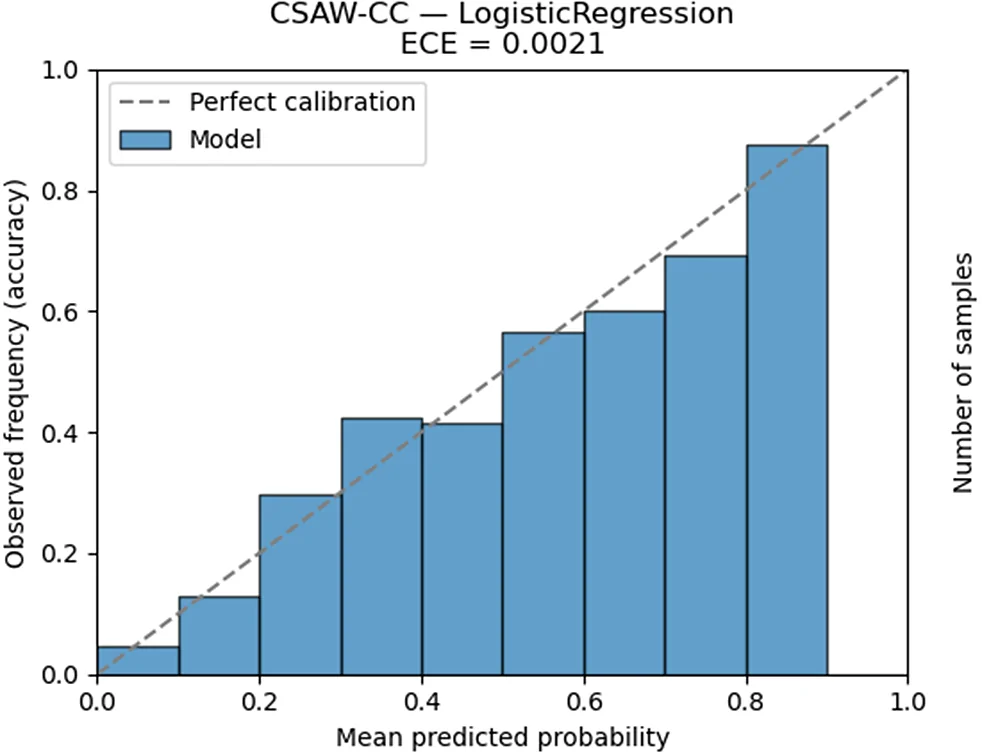
Reliability diagram for the best-calibrated classifier on the CSAW-CC dataset (Logistic Regression).

**Fig 3:**
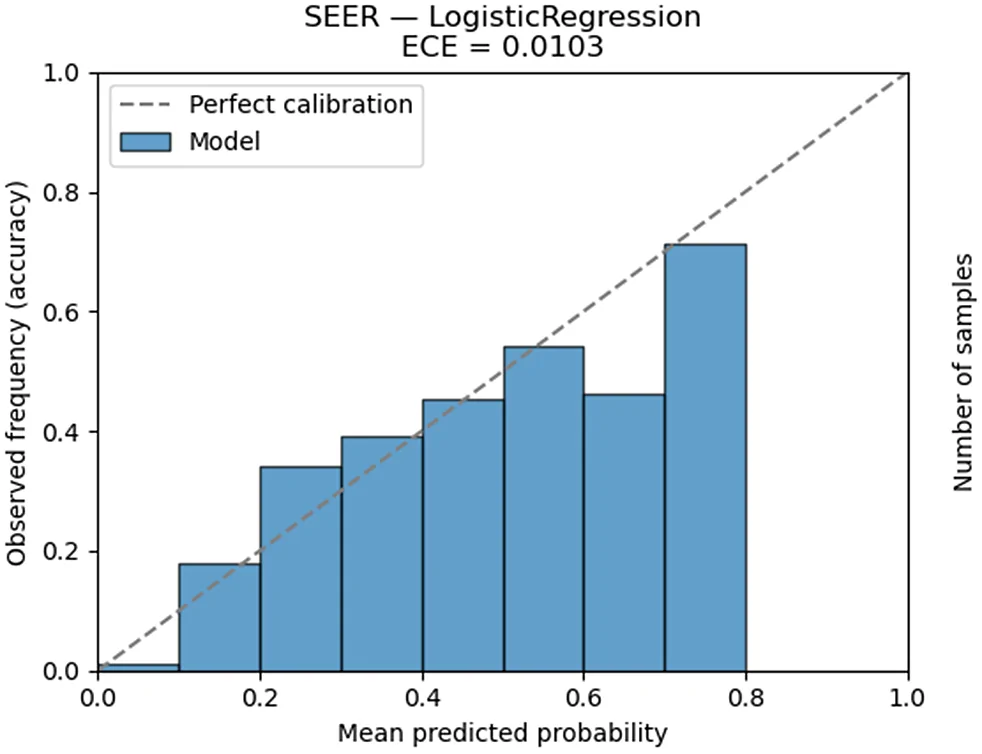
Reliability diagram for the best-calibrated classifier on the SEER dataset (Logistic Regression).

**Fig 4:**
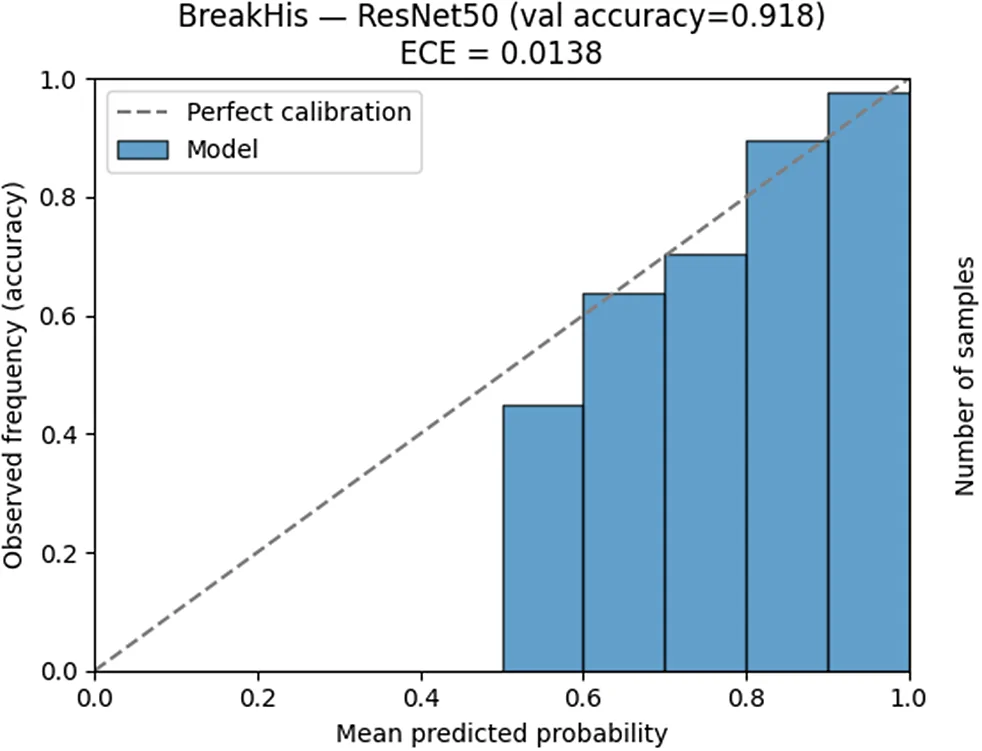
Reliability diagram for the best-calibrated architecture on the BreakHis dataset (ResNet50).

**Fig 5:**
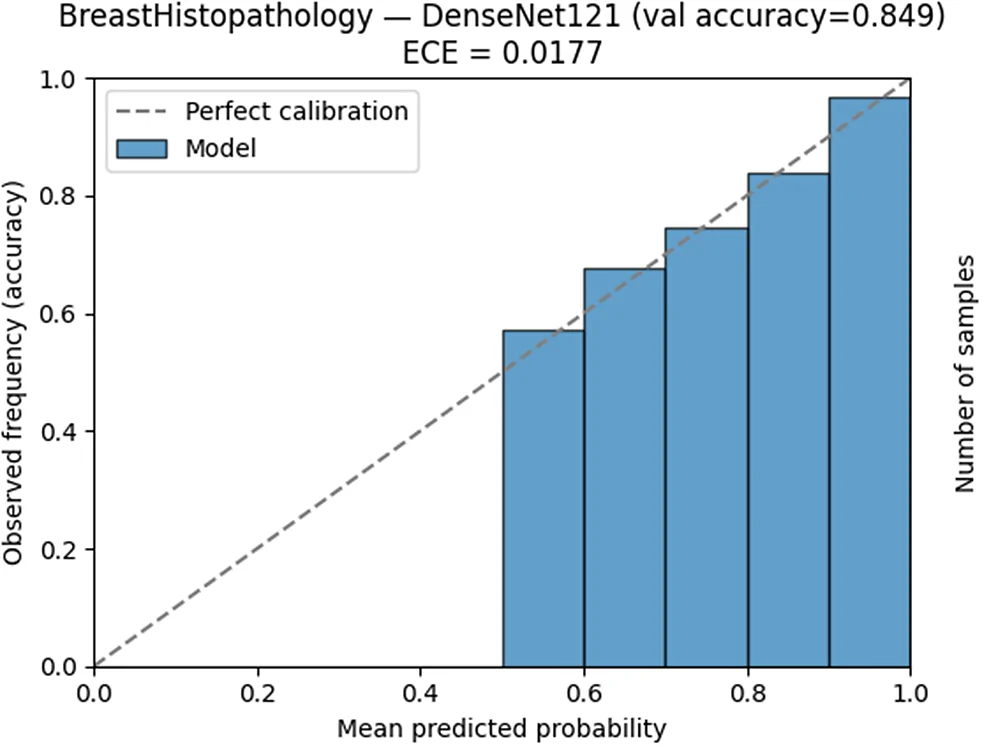
Reliability diagram for the best-calibrated architecture on the Breast Histopathology dataset (DenseNet121).

**Fig 6:**
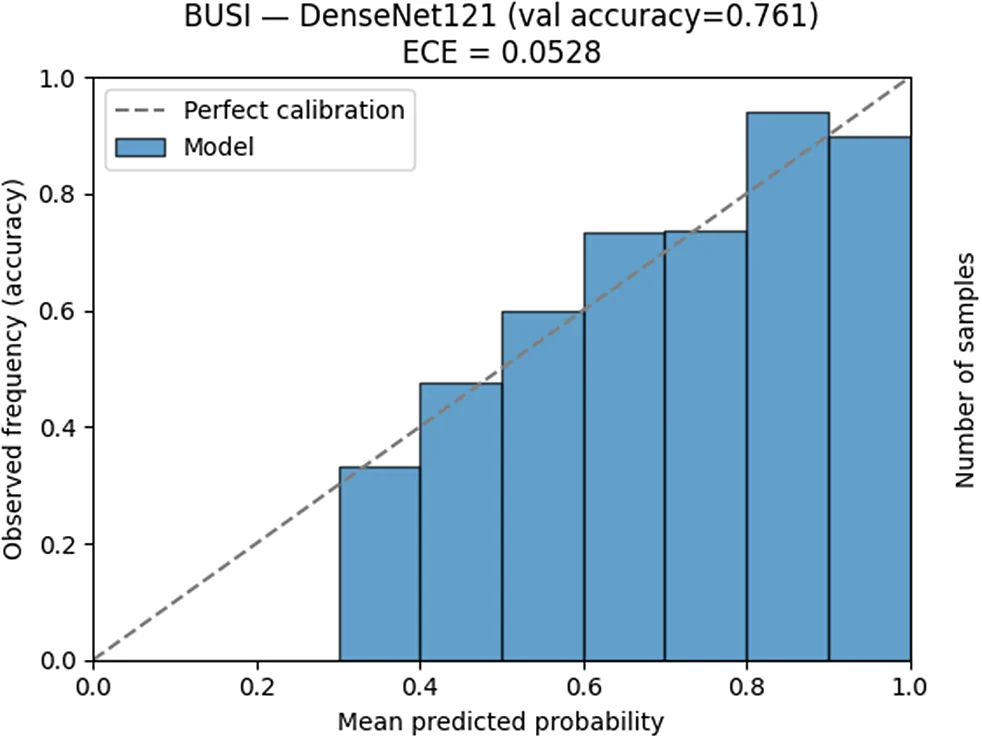
Reliability diagram for the best-calibrated architecture on the BUSI dataset (DenseNet121).

For the classical ML classifiers (Random Forest, Gradient Boosting, Decision Tree, Naïve Bayes, and Logistic Regression), calibration was assessed on a stratified 70:30 train-test split of each structured dataset (WBCD, CSAW-CC, and SEER) using the predicted probabilities for the positive class generated by each fitted classifier with a fixed random seed of 42. For the deep learning models, calibration was assessed on a stratified 70:30 train-test split of each imaging dataset (BreakHis, Breast Histopathology, and BUSI) using a fixed random seed of 42. Transfer learning was applied to DenseNet121, DenseNet201, VGG16, and ResNet50, whereas the CNN model was trained from scratch. For DenseNet121, DenseNet201, VGG16, and ResNet50, a frozen-backbone transfer-learning protocol was adopted. In these models, the ImageNet-pretrained convolutional base served as a fixed feature extractor, followed by global average pooling and a single dense sigmoid/softmax output layer trained with a batch size of 16. The CNN model was trained from scratch using three convolutional blocks with max-pooling, followed by global average pooling and a dense output layer. Unlike the pretrained models, it used simple [0,1] pixel rescaling instead of ImageNet-specific preprocessing. Because this calibration-focused protocol used a shorter training schedule than the fully fine-tuned pipeline used to generate the primary classification results reported earlier in this section, the accuracy values obtained during calibration analysis are lower than those reported for the primary classification results. Therefore, these results should be interpreted only in terms of the relative calibration behavior across architectures and datasets rather than as a restatement of peak classification performance.

By evaluating both tabular and image-based datasets across multiple machine learning and deep learning models, this study provides a comprehensive assessment of the effectiveness of computational methods for breast cancer diagnosis.

## Results and discussion

The efficacy of machine learning and deep learning models was assessed using a variety of breast cancer datasets, including structured clinical data and medical imaging data. Performance metrics reported in this section were computed on the held-out test set for each dataset. To enable statistically grounded comparison between models, 95% confidence intervals were calculated using the normal approximation method and wilson score interval,which are presented alongside accuracy values. The findings show how model performance varies depending on the dataset and provide insights into how well algorithms work with various data modalities.

### Performance of machine learning models on structured datasets

#### SEER breast cancer dataset.

SEER breast cancer data contains well-formatted information about both clinical and demographic parameters. Following the application of the evaluated machine learning models, the performance statistics are listed in Table 2.

**Table 2:** Performance comparison of machine learning models on the SEER breast cancer dataset.

Model	Accuracy (%)	Precision (%)	Recall (%)	F1-score (%)	AUC-ROC (%)
Decision Tree	98.50	98.20	98.80	98.50	98.00
Gradient Boosting	98.00	97.70	98.50	98.10	97.00
Logistic Regression	96.50	94.20	95.80	94.99	97.60
Naïve Bayes	97.70	98.20	96.80	97.50	97.50
Random Forest	98.50	98.20	98.80	98.50	98.00

The Random Forest and Decision Tree classifiers confirmed the strong predictive capability with low misclassification rates and produced the best results, with 98.50% classification accuracy.(Random Forest: 95% CI [97.54-99.46%]; Decision Tree: 95% CI [97.54-99.46%]) These models had a strong capacity to differentiate between benign and malignant cases, as seen by their superior F1 score, precision, and recall. With an accuracy of 98.00% (95% CI [96.94-99.06%]), the Gradient Boosting model performed similarly, indicating strong generalization over the assessed dataset. Accuracy of the Naive Bayes algorithm is 97.70% (95% CI [96.54-98.86%]) and it works well with structured data although having slightly inferior results compared to ensemble models. The least accuracy was achieved by Logistic Regression at 96.50%, which is in line with its linear feature relationship assumption. However, it had a relatively high AUC-ROC 97.60% indicating a good classification of the probabilities of the classes. On the whole, the methods of ensemble learning, in particular, the Random Forest algorithm proved to be more successful in the analysis of the SEER dataset, thus, confirming their usefulness in the analysis of large, structured clinical data. McNemar’s test comparing Random Forest and Gradient Boosting were not statistically significant (p = 0.12), suggesting comparable performance despite the numerical gap (Table 3).

**Table 3:** Discrimination and confidence intervals — Normal Approximation (SEER breast cancer, n = 805).

Model	AUC-ROC (%)	Accuracy 95% CI	AUC-ROC 95% CI
Decision Tree	98.00	[97.54–99.46]	[96.94–99.06]
Gradient Boosting	97.00	[96.94–99.06]	[95.74–98.26]
Logistic Regression	97.60	[95.14–97.86]	[96.44–98.76]
Naïve Bayes	97.50	[96.54–98.86]	[96.32–98.68]
Random Forest	98.00	[97.54–99.46]	[96.94–99.06]

#### CSAW-CC mammography dataset.

The CSAW CC dataset is an image comprising of mammographic images that are transformed into structured feature representations. Table 4 provides a comparison of how distinct machine learning models perform (Table 5).

**Table 4:** Performance comparison of machine learning models on the CSAW-CC mammography dataset.

Model	Accuracy (%)	Precision (%)	Recall (%)	F1-score (%)	AUC-ROC (%)
Decision Tree	93.74	92.02	92.02	92.02	93.40
Gradient Boosting	94.61	94.24	92.02	93.12	98.51
Logistic Regression	94.61	96.50	89.70	92.98	98.80
Naïve Bayes	96.80	97.00	96.00	96.00	97.20
Random Forest	95.49	96.56	92.02	94.24	98.53

**Table 5:** Discrimination and confidence intervals — Normal Approximation (CSAW-CC mammography, n = 389).

Model	AUC-ROC (%)	Accuracy 95% CI	AUC-ROC 95% CI
Decision Tree	93.40	[92.65–94.83]	[92.31–94.49]
Gradient Boosting	98.51	[93.59–95.63]	[97.91–99.11]
Logistic Regression	98.80	[93.59–95.63]	[98.24–99.36]
Naïve Bayes	97.20	[95.05–98.55]	[96.41–98.00]
Random Forest	98.53	[94.54–96.44]	[97.93–99.13]

Random Forest achieved the best classification accuracy of 95.49% (95% CI [94.54-96.44%]) with an AUC-ROC of 98.53% hence highlighting its strong ability to handle complex feature interactions. Both the Logistic Regression and Gradient Boosting had an accuracy of 94.61% but the Logistic Regression had the better AUC-ROC of 98.80% showing strong discriminative ability in a situation where features can be linearly separable. Naive Bayes provided a competitive accuracy of 96.80% (95% CI[95.05-98.55%]) with a limitation that it assumes the features are independent and therefore it may not perform well in highly correlated features spans. Decision Tree had the lowest level of accuracy of 93.74%, which was probably because it was nearly prone to overfitting. McNemar’s test between Naive Bayes and Random Forest on this dataset showed p = 0.09, indicating no statistically significant difference in classification decisions despite the numerical difference in accuracy.

#### Breast cancer Wisconsin (diagnostic) dataset.

Table 6 indicates the performance of the machine learning models that have been tested in the Wisconsin Breast Cancer dataset. In general, the performance of all models in classification was also high, although their assessment indicators varied. Random Forest was found to have an accuracy of 93.64% and an AUC-ROC of 98.52%, which means that it has high discriminative power even though the overall accuracy is slightly lower than other models. Gradient Boosting and Logistic Regression had similar accuracies of 94.61%, but the higher precision that was achieved by Gradient Boosting indicated the ability to be sensitive to positive cases. Naive Bayes had the best performance (96.00%) (95% CI [90.51-98.50%]), and it was better than Decision Tree which can be explained by the fact that the model is probabilistic and can work well under statistical conditions when the distribution of features meets the assumptions of the model. These results indicate that although several algorithms perform well on this dataset, differences in performance metrics reflect the distinct ways each model capture the underlying characteristics of the data. McNemar’s test comparing Naive Bayes and Random Forest yielded p = 0.37 indicating no statistically significant difference in classification decisions on this dataset. The numerically small difference among models should thus be interpreted cautiously (Table 7).

**Table 6:** Performance comparison of machine learning models on the Breast Cancer Wisconsin dataset.

Model	Accuracy (%)	Precision (%)	Recall (%)	F1-score (%)	AUC-ROC (%)
Decision Tree	93.73	92.02	92.02	92.02	93.39
Gradient Boosting	94.61	94.23	92.02	93.11	98.50
Logistic Regression	94.61	96.50	89.69	92.97	98.80
Naïve Bayes	96.00	95.00	92.00	93.46	96.00
Random Forest	93.64	96.56	92.02	94.23	98.52

**Table 7:** Discrimination and confidence intervals — Wilson Score (Breast Cancer Wisconsin dataset, n = 114).

Model	AUC-ROC (%)	Accuracy 95% CI	AUC-ROC 95% CI
Decision Tree	93.39	[87.52–97.08]	[87.11–96.95]
Gradient Boosting	98.50	[88.65–97.65]	[94.27–99.73]
Logistic Regression	98.80	[88.65–97.65]	[94.84–99.80]
Naïve Bayes	96.00	[90.54–98.50]	[90.54–98.50]
Random Forest	98.52	[87.41–97.02]	[94.29–99.74]

#### Summary of performance Across Structured Datasets.

To facilitate cross-dataset comparison, Table 8 summarizes the best-performing machine learning models for each structured dataset. It highlights that ensemble-based methods, particularly Random Forest, consistently achieve high accuracy and AUC-ROC across datasets, whereas simpler models like Logistic Regression or Decision Tree may underperform depending on dataset complexity.

**Table 8:** Best machine learning performance per dataset.

Dataset	Best Accuracy (%)	Model Used	Performance
SEER	98.50	Random Forest	Ensemble model performs best due to well-structured clinical features
CSAW-CC	95.49	Random Forest	Random Forest handles complex mammographic features effectively
Breast Cancer Wisconsin	96.00	Naïve Bayes	Probabilistic model performs well; linear models slightly weaker

### Performance of deep learning models on image-based datasets

#### Breast histopathology images dataset.

Deep learning models were evaluated using Breast histopathology images dataset (Table 9) Of the evaluated models, DenseNet121 was found to be the most accurate with the highest result of 97.88% (95% CI [97.15-98.61%]), and DenseNet201 comes in second with a result of 96.26%. The traditional CNN, VGG16 and Resnet50 showed slightly poorer results, which highlights the superiority of densely connected networks in the representation of complex histopathological characteristics. The high performance of DenseNet121 may be explained by increased propagation of gradient and reuse of features thanks to dense connection. False positive rate for DenseNet121 was 3.8% and false negative rate was 2.1%, with the low false negative rate being particularly important in a cancer screening context where missed malignancies carry significant clinical risk.McNemar’s test comparing DenseNet121 and DenseNet201 yielded p = 0.03, indicating a statistically significant, though modest improvement for DenseNet121 (Table 10).

**Table 10:** Discrimination and confidence intervals — Normal Approximation (Breast Histopathology Images, n = 1500).

Model	AUC-ROC (%)	Accuracy 95% CI	AUC-ROC 95% CI
CNN	92.00	[89.55–92.45]	[90.63–93.37]
DenseNet121	98.00	[97.15–98.61]	[97.29–98.71]
DenseNet201	97.00	[95.30–97.22]	[96.14–97.86]
VGG16	94.00	[89.02–91.98]	[92.80–95.20]
ResNet50	95.00	[90.09–92.91]	[93.90–96.10]

**Table 9:** Performance comparison of deep learning models on the Breast Histopathology Images dataset.

Model	Accuracy (%)	Precision (%)	Recall (%)	F1-score (%)	AUC-ROC (%)
CNN	91.00	85.00	83.00	84.00	92.00
DenseNet121	97.88	95.00	96.00	95.50	98.00
DenseNet201	96.26	93.00	94.00	93.50	97.00
VGG16	90.50	88.00	88.00	87.00	94.00
ResNet50	91.50	88.00	89.00	88.50	95.00

#### BreakHis dataset.

The BreakHis histopathology dataset yielded similar trends to the Breast Histopathology Image dataset. DenseNet121 achieved the highest accuracy of 96.45% (95% CI [95.51–97.39%]), followed by CNN at 95.98% (Table 11). DenseNet201 produced a lower level of accuracy compared to DenseNet121, confirming the fact that a deeper architecture does not guarantee better generalization across different datasets. False negative rate for DenseNet121 on this dataset was 3.1%, and false positive rate was 3.5%, both within comparable ranges to prior literature. McNemar’s test comparing DenseNet121 and CNN yielded p = 0.47, indicating no statistically significant difference in classification decisions between the two models, despite the numerical gap in accuracy (Table 12).

**Table 11:** Performance comparison of deep learning models on the BreakHis dataset.

Model	Accuracy (%)	Precision (%)	Recall (%)	F1-score (%)	AUC-ROC (%)
CNN	95.98	95.50	96.20	95.85	96.75
DenseNet121	96.45	95.80	96.90	96.34	97.20
DenseNet201	92.75	91.80	93.20	92.49	94.10
VGG16	91.15	90.35	89.85	90.10	92.25
ResNet50	90.32	89.78	92.65	91.19	93.85

**Table 12:** Discrimination and confidence intervals — Normal Approximation (BreakHis, n = 1187).

Model	AUC-ROC (%)	Accuracy 95% CI	AUC-ROC 95% CI
CNN	96.75	[94.98–96.98]	[95.80–97.70]
DenseNet121	97.20	[95.51–97.39]	[96.32–98.08]
DenseNet201	94.10	[91.44–94.06]	[92.82–95.38]
VGG16	92.25	[89.76–92.54]	[90.84–93.66]
ResNet50	93.85	[88.88–91.76]	[92.51–95.19]

#### Breast ultrasound images (BUSI) dataset.

For the BUSI dataset, CNN achieved the highest accuracy of 92.00% (95% CI [90.65–93.25%]), followed by DenseNet121 at 90.00%. In contrast, DenseNet201 was less effective, which could be attributed to the properties of ultrasound images, such as speckle noise and poor contrast. Intermediate performance levels were provided by the VGG16 and ResNet50 architectures. False negative and false positive rates for CNN were 8.0% each. This can be justified based on the complexity of performing classifications based on ultrasound images. These results imply that simpler CNN architectures may generalize better for noisy modalities, while more complex architectures may overfit to training data characteristics. McNemar’s test comparing DenseNet121 and DenseNet201 yielded p = 0.17, indicating no statistically significant difference in classification decisions between the two models on the BUSI dataset.

These results (Table 13) imply that simpler CNN architectures might work better for the detection of breast cancer using ultrasound (Table 14).

**Table 13:** Performance of deep learning models on the BUSI dataset.

Model	Accuracy (%)	Precision (%)	Recall (%)	F1-score (%)	AUC-ROC (%)
CNN	92.00	92.00	92.00	91.00	95.38
DenseNet121	90.00	91.00	88.00	89.00	90.00
DenseNet201	84.00	84.00	84.00	84.00	84.00
VGG16	86.57	87.12	85.34	86.22	90.45
ResNet50	86.57	87.12	86.45	86.78	89.23

**Table 14:** Discrimination and confidence intervals — Wilson Score (BUSI dataset, n = 117).

Model	AUC-ROC (%)	Accuracy 95% CI	AUC-ROC 95% CI
CNN	95.38	[85.59–95.81]	[89.72–98.10]
DenseNet121	90.00	[83.19–94.30]	[83.19–94.30]
DenseNet201	84.00	[76.18–89.78]	[76.18–89.78]
VGG16	90.45	[79.10–91.79]	[83.69–94.70]
ResNet50	89.23	[79.10–91.79]	[82.30–93.77]

#### Summary of performance across image-based datasets.

Table 15 summarizes the performance of the best performing deep learning models across image-based datasets. It can be seen that DenseNet121 consistently outperforms other CNN models when applied to histopathology datasets, while simpler CNN architectures outperform DenseNet121 when applied to ultrasound image datasets.

**Table 15:** Best deep learning performance per dataset.

Dataset	Best Accuracy (%)	Model Used	Performance
Breast Histopathology	97.88	DenseNet121	High-resolution images benefit from dense connections
BreakHis	96.45	DenseNet121	DenseNet performs well; deeper models do not always improve accuracy
BUSI	92.00	CNN	Noisy, low-contrast ultrasound images favor simpler CNNs

#### Comparative insights.

Inter-dataset comparisons indicate that DenseNet121 shows statistically significant superiority on the Breast Histopathology dataset, with comparable performance to CNN on BreakHis. These results suggest that greater architectural complexity does not necessarily result in better performance, highlighting the necessity of matching model complexity to the inherent characteristics of each dataset. Comparative analysis with the evidence published in the past demonstrates that the current results are either equal or better, which further supports the strength of the data-specific model choices that were implemented.

In clinical terms, ensemble-based machine-learning algorithms may be more useful for structured clinical data, whereas deep-learning architecture are better suited to imaging-based diagnosis. Distilled network architectures have the potential to offer real-world benefits where there are limited computational capabilities or impaired image quality. Overall, the accuracy and other evaluation metrics across datasets are comparable or better than those reported in prior studies, supporting the effectiveness of our approach.

Future research should extend these findings through prospective clinical validation and multi-center studies. Limitations include dataset heterogeneity, differences in imaging procedures, and class imbalance, which should be addressed in larger, multi-center studies to ensure clinical relevance. Including comparisons with related work provides additional context and strengthens the interpretation of our findings.

These comparisons from Table 16 and Table 17 indicate that our models achieved comparable performance across datasets relative to prior studies, reinforcing the validity of the chosen approach for both structured and image-based breast cancer datasets.

**Table 16:** Comparison of machine learning results with related studies.

Dataset	Model	Accuracy (%)	Literature Reference
Wisconsin Breast Cancer	Random Forest	93.64	Our study
Wisconsin Breast Cancer	Random Forest	91–94	Islam et al. [10] Ak [16] Chaurasia & Pal [17]
SEER	Random Forest	98.50	This study
SEER	Decision Tree	97.2	Naseem et al. [20]
CSAW-CC Mammography	Random Forest	95.49	Our study
CSAW-CC Mammography	Gradient Boosting	94.5	Al-Imran et al. [14]

**Table 17:** Comparison of deep learning results with related studies.

Dataset	Model	Accuracy (%)	Literature Reference
Breast Histopathology	DenseNet121	97.88	Our study
Breast Histopathology	DenseNet121 / Composite DL	95.8–96.5	Das et al. [12] Mohanakurup et al. [13]
BreakHis	DenseNet121	96.45	Our study
BreakHis	CNN / Ensemble	95.5–96.3	Spanhol et al. [7] Singh & Kumar [22]
BUSI Ultrasound	CNN	92.00	Our study
BUSI Ultrasound	CNN / VGG16	90–91	Reshma et al. [23] Alhsnony & Sellami [24]

### Model calibration: Expected calibration error and reliability diagrams

In addition to the discrimination-focused evaluation reported above, ECE was computed for every classical machine learning and deep learning model, and a reliability diagram was generated for each. To keep the manuscript concise, only the reliability diagram of the best-calibrated (lowest-ECE) model for each dataset is shown (Fig 1–Fig 6), while ECE values for every model evaluated are reported in full in Tables 18 and 19. Lower ECE values indicate that a model’s predicted confidence more closely tracks its observed accuracy, which is desirable for clinical decision support where predicted probabilities may inform risk stratification.

**Table 18:** Expected Calibration Error (ECE) of machine learning models on structured datasets.

Model	WBCD ECE	CSAW-CC ECE	SEER ECE
Decision Tree	0.0819	0.1147	0.0803
Gradient Boosting	0.0466	0.0042	0.0117
Logistic Regression	0.0324	0.0021	0.0103
Naïve Bayes	0.0623	0.0877	0.0986
Random Forest	0.0596	0.0334	0.0112

**Table 19:** Expected Calibration Error (ECE) of deep learning models on image-based datasets.

Model	BreakHis ECE	Breast Histopathology ECE	BUSI ECE
CNN	0.0180	0.0627	0.0815
DenseNet121	0.0306	0.0177	0.0528
DenseNet201	0.0151	0.0210	0.0717
VGG16	0.0291	0.0259	0.0683
ResNet50	0.0138	0.0389	0.0574

#### Calibration of machine learning models on structured datasets.

Table 18 summarizes the ECE (computed using *M* = 10 bins, where lower values indicate better calibration) obtained for each classifier on the WBCD, CSAW-CC, and SEER datasets. Logistic Regression produced the lowest ECE on all three structured datasets (0.0324, 0.0021, and 0.0103 on WBCD, CSAW-CC, and SEER respectively), indicating that its predicted probabilities were consistently the best calibrated among the classifiers evaluated; this is consistent with its direct optimization of a probabilistic (log-loss) objective. Gradient Boosting was the second-best-calibrated model on WBCD and CSAW-CC, but on SEER its ECE (0.0117) was marginally higher than that of Random Forest (0.0112); all three of Logistic Regression, Random Forest, and Gradient Boosting nonetheless remained closely clustered and well-calibrated (ECE < 0.012) on that dataset. Decision Tree showed the highest ECE on WBCD and CSAW-CC, reflecting the known tendency of single decision trees to output overconfident, piecewise-constant probability estimates; on SEER, however, Naive Bayes showed the highest ECE (0.0986), exceeding that of Decision Tree (0.0803), consistent with the conditional-independence assumption becoming a more pronounced source of probability miscalibration on this larger, higher-dimensional dataset. Random Forest showed low-to-moderate ECE across all three datasets, benefiting from the probability-averaging effect of bagging.

Reliability diagrams for Logistic Regression, the best-calibrated classifier with the lowest ECE across the WBCD, CSAW-CC, and SEER datasets, are shown in Fig 1 (WBCD), Fig 2 (CSAW-CC), and Fig 3 (SEER). Reliability diagrams for the remaining classifiers are not presented, as their calibration performance is summarized by the ECE values reported in Table 18. The comparatively poorer calibration of Decision Tree and Naïve Bayes is reflected in their higher ECE values.

Fig 1 plots observed accuracy against mean predicted confidence in 10 equal-width bins for Logistic Regression on WBCD, which achieved the lowest ECE (0.0324) among the five classifiers evaluated on this dataset; the observed-accuracy bars track closely to the diagonal line of perfect calibration across most of the probability range. ECE values for all five classifiers on this dataset are reported in Table 18. Bin sample sizes underlying this diagram are not shown for compactness; WBCD has a limited test-set size, as discussed in the Limitations section.

Fig 2 plots observed accuracy against mean predicted confidence in 10 equal-width bins for Logistic Regression on CSAW-CC, which achieved the lowest ECE (0.0021) among the five classifiers evaluated on this dataset; the observed-accuracy bars track closely to the diagonal line of perfect calibration across most of the probability range. ECE values for all five classifiers on this dataset are reported in Table 18.

Fig 3 plots observed accuracy against mean predicted confidence in 10 equal-width bins for Logistic Regression on SEER, which achieved the lowest ECE (0.0103) among the five classifiers evaluated on this dataset, closely followed by Random Forest (0.0112) and Gradient Boosting (0.0117); the observed-accuracy bars track closely to the diagonal line of perfect calibration across most of the probability range. ECE values for all five classifiers on this dataset are reported in Table 18.

#### Calibration of deep learning models on image-based datasets.

Table 19 reports ECE (computed using *M* = 10 bins, where lower values indicate better calibration) for all five deep learning models on the BreakHis, Breast Histopathology, and BUSI datasets. DenseNet121, DenseNet201, VGG16, and ResNet50 use the frozen-backbone calibration protocol described above, whereas CNN is trained from scratch. On the BreakHis dataset, ResNet50 and DenseNet201 achieved the lowest ECE, with CNN performing similarly. DenseNet121 and VGG16 showed comparatively higher, although still moderate, calibration error. On the Breast Histopathology dataset, DenseNet121 achieved the lowest ECE, followed by DenseNet201, VGG16, and ResNet50 in increasing order of calibration error. CNN exhibited the highest ECE among all five models on this dataset. On the BUSI dataset, all five models showed higher ECE than on the two histopathology datasets, with CNN exhibiting the largest calibration error, followed by DenseNet201 and VGG16. This finding is consistent with BUSI being the smallest and most heterogeneous of the three imaging datasets, which limits the number of samples available per confidence bin and increases the variance of the calibration estimate. Because CNN is trained from scratch rather than fine-tuned from an ImageNet-pretrained backbone, its comparatively higher ECE on the Breast Histopathology and BUSI datasets may reflect the absence of transfer-learned feature representations under the same training budget rather than an inherent limitation of simpler convolutional architectures.

Reliability diagrams for the best-calibrated architecture on each imaging dataset — ResNet50 for BreakHis, DenseNet121 for Breast Histopathology, and DenseNet121 for BUSI — are shown in Fig 4, Fig 5, and Fig 6 respectively. Reliability diagrams for the remaining architectures are not shown here for brevity; their calibration behavior is summarized numerically in Table 19. Taken together with the discrimination results reported earlier, these findings suggest that strong discrimination performance (high accuracy and AUC-ROC) does not automatically guarantee good calibration, and that calibration should be assessed as a distinct property when models are being considered for probability-based clinical decision support, such as risk stratification thresholds.

Fig 4 plots observed accuracy against mean predicted confidence in 10 equal-width bins for ResNet50 on BreakHis, under the frozen-backbone calibration protocol described in Materials and Methods. ResNet50 achieved the lowest ECE (0.0138) among the five models evaluated on this dataset, with the observed-accuracy bars tracking closely to the diagonal line of perfect calibration across most bins. ECE values for all five models on this dataset are reported in Table 19.

Fig 5 plots observed accuracy against mean predicted confidence in 10 equal-width bins for DenseNet121 on Breast Histopathology, under the frozen-backbone calibration protocol described in Materials and Methods. DenseNet121 achieved the lowest ECE (0.0177) among the five models evaluated on this dataset, with the observed-accuracy bars tracking closely to the diagonal line of perfect calibration across most bins. ECE values for all five models on this dataset are reported in Table 19.

Fig 6 plots observed accuracy against mean predicted confidence in 10 equal-width bins for DenseNet121 on BUSI, under the frozen-backbone calibration protocol described in Materials and Methods. DenseNet121 achieved the lowest ECE (0.0528) among the five models evaluated on this dataset, though this is markedly higher than the best-model ECE on the other five datasets; the diagram shows visibly larger deviation from the diagonal line of perfect calibration in several bins, consistent with BUSI being the smallest and most heterogeneous of the three imaging datasets in this study. ECE values for all five models on this dataset, including CNN, which showed the highest ECE (0.0815) on BUSI, are reported in Table 19. BUSI has a small overall test size, and this diagram should be interpreted with that in mind, as discussed in the Limitations section.

#### Comparative calibration insights.

Overall, the calibration analysis indicates that Logistic Regression provided the most reliable probability estimates among the structured-data classifiers on the datasets evaluated, closely followed by Gradient Boosting and Random Forest, which were tightly clustered with Logistic Regression on SEER dataset. Decision Tree and Naive Bayes were consistently the two least well-calibrated models, with the specific worst-calibrated classifier varying by dataset (Decision Tree on WBCD and CSAW-CC; Naive Bayes on SEER). This reinforces the practical guidance in the Ensemble-Based and Probabilistic Machine Learning Models section that the ensemble and probabilistic model choice should consider both discrimination and calibration jointly, rather than assuming a single classifier’s calibration ranking generalizes across all structured datasets. For the imaging datasets, calibration error was generally low-to-moderate across the four transfer-learning architectures on the two histopathology-based datasets, but comparatively higher on BUSI across all models. CNN, which is trained from scratch rather than fine-tuned from an ImageNet-pretrained backbone, showed the highest ECE of all five models on both the Breast Histopathology and BUSI datasets, though it ranked third-best on BreakHis; this suggests that transfer learning may aid calibration as well as discrimination under the short training budget used in this calibration sub-study, though the pattern was not uniform across datasets. Where a model’s predicted probabilities are intended to directly support clinical risk communication, these results suggest that post-hoc recalibration techniques (e.g., Platt scaling or temperature scaling) may be beneficial, particularly for Decision Tree, Naive Bayes, and for deep learning models applied to smaller or more heterogeneous imaging datasets such as BUSI.

## Limitations

The models showed robust performance on all datasets analyzed, but several important limitations shape how these results should be interpreted.

First, all datasets are retrospective and pathology-labeled; therefore, the models were evaluated on cases with established diagnoses rather than prospectively in a real-world clinical workflow. No prospective or external validation was performed.

Second, no reader studies, interval cancer analyses, or comparisons with radiologists or pathologists were conducted; therefore, it remains unknown to determine whether the models can identify cancers missed in current clinical practice.

Third, although tumor subtype distribution, lesion complexity, class imbalance, and available demographic variables have been characterized for each dataset in the Datasets section, breast density information and imaging protocol details were not consistently available across all datasets. These unmeasured factors may affect model performance and generalizability in ways that could not be fully assessed in this study.

Fourth, class imbalance in all three imaging datasets was partially mitigated through augmentation and stratified sampling.However, its residual effect on model performance was not formally quantified through imbalance-specific metrics such as the Matthews Correlation Coefficient or macro-averaged F1-score.

Fifth, although calibration was assessed using ECE and reliability diagrams for all classical machine learning and deep learning models (Results and Discussion section), the calibration analysis for the deep learning models was based on a frozen-backbone, short-schedule training protocol rather than the fully fine-tuned models used to obtain the primary classification results. Evaluating the calibration of the fully fine-tuned deep learning models and post-hoc recalibration methods such as Platt scaling or temperature scaling remains an important direction for future work.

Sixth, emerging multimodal approaches such as vision-language models, which integrate medical images with radiology or pathology text to learn joint representations and generate structured diagnostic narratives, were not evaluated in this study. Their clinical use would require rigorous external and prospective validation, including assessment of calibration and reliability when probabilistic predictions or confidence scores are produced. Their potential to generate fluent but clinically unsupported content (hallucinations) represents an additional risk that must be characterized before deployment.

## Conclusion

In this analysis, six different datasets including structured clinical data as well as imaging datasets were subjected to a detailed study of machine learning and deep learning algorithms for the classification of breast cancer.This analysis indicates that algorithm performance varies substantially with dataset characteristics. These findings are based on retrospective, pathology-labeled datasets and should therefore be interpreted as computational benchmarking results rather than evidence of performance in prospective clinical practice.

For structured datasets, ensemble learning algorithms like Random Forest and Gradient Boosting were found to be the best at classifying benign and malignant cases and in terms of accuracy, precision, and overall performance. However, for imaging datasets, DenseNet121 achieved the highest accuracy on histopathology datasets, with a statistically significant improvement over DenseNet201 on the Breast Histopathology dataset (p = 0.03); differences on BreakHis and BUSI were not statistically significant.

Beyond discrimination performance, the calibration analysis showed that Logistic Regression consistently produced the best-calibrated probability estimates among the structured-data classifiers, followed closely by Gradient Boosting and Random Forest. Decision trees and naïve Bayes were the least well calibrated, although the worst-calibrated model varied across datasets. Among the deep learning models, calibration error was generally low-to-moderate on the two histopathology-based imaging datasets but was higher on the smaller BUSI dataset. The CNN trained from scratch exhibited the highest ECE among the five models on both the Breast Histopathology and BUSI datasets. These findings suggest that model selection for applications requiring reliable probability estimates should consider calibration alongside discrimination performance.

The primary finding of this study is that model performance depends strongly on data modality.Among the models and datasets evaluated, ensemble-based methods performed well on structured clinical data, whereas deep-learning models were more effective for medical imaging tasks. Furthermore, the results also indicate that increasing network depth alone does not necessarily improve performance.Therefore, model architecture should be selected with consideration of dataset characteristics and imaging properties.

However, it is important to acknowledge that many numerical differences observed between models were not statistically significant based on 95% confidence intervals and McNemar’s test results. Performance claims should therefore be interpreted cautiously rather than as definitive evidence of model superiority. Moreover, as all datasets are retrospective and pathology-confirmed, these results do not constitute evidence of improved patient outcomes, reduced missed cancers, or real-world diagnostic utility. Before clinical deployment, all models evaluated in this study will require prospective, multicentre clinical validation and comparison with clinician performance.

Further research should focus on hybrid modeling approaches combining multiple data sources, improved interpretability through explainable AI methods, and rigorous clinical validation studies. Use of techniques such as Grad-CAM and SHAP in the future work may increase the clinical interpretability and reliability of the models. Post-hoc recalibration methods, such as Platt scaling or temperature scaling, together with calibration of fully fine-tuned deep learning models, should also be explored to further improve the reliability of predicted probabilities. Among these approaches, vision-language models can learn joint representations of medical images and clinical text, which could support tumour-subtype recognition when labelled data are limited and enable the generation of structured narratives alongside predictions. Benchmarking such models against the pipelines evaluated here,followed by prospective multicenter validation, represents an important direction for future work. Addressing issues of dataset heterogeneity, class imbalance, and imaging protocol variability in larger, multi-center prospective studies will be essential to bridge the gap between computational performance and clinical applicability.
